# PTPRD and CNTNAP2 as markers of tumor aggressiveness in oligodendrogliomas

**DOI:** 10.1038/s41598-022-14977-2

**Published:** 2022-08-18

**Authors:** Kirsi J. Rautajoki, Serafiina Jaatinen, Aliisa M. Tiihonen, Matti Annala, Elisa M. Vuorinen, Anni Kivinen, Minna J. Rauhala, Kendra K. Maass, Kristian W. Pajtler, Olli Yli-Harja, Pauli Helén, Joonas Haapasalo, Hannu Haapasalo, Wei Zhang, Matti Nykter

**Affiliations:** 1 Prostate Cancer Research Center, Faculty of Medicine and Health Technology, Tampere University and Tays Cancer Center, Tampere University Hospital, Tampere, Finland; 2 Tampere Institute for Advanced Study, Tampere University, Tampere, Finland; 3 Department of Neurosurgery, Tampere University Hospital, Tampere, Finland; 4 Faculty of Medicine and Health Technology, Tampere University and Tays Cancer Center, Tampere, Finland; 5 Hopp Children’s Cancer Center Heidelberg (KiTZ), Heidelberg, Germany; 6 Division of Pediatric Neuro Oncology, German Cancer Consortium (DKTK), German Cancer Research Center, Heidelberg, Germany; 7 Department of Pediatric Oncology, Hematology, Immunology and Pulmonology, Heidelberg University Hospital, Heidelberg, Germany; 8 Institute for Systems Biology, Seattle, WA USA; 9 Fimlab Laboratories Ltd., Tampere University Hospital, Tampere, Finland; 10 Cancer Genomics and Precision Oncology, Wake Forest Baptist Comprehensive Cancer Center, Winston-Salem, NC USA; 11 Foundation for the Finnish Cancer Institute, Helsinki, Finland

**Keywords:** Next-generation sequencing, RNA sequencing, Cancer genomics, CNS cancer, Tumour biomarkers, Cancer genetics, Chromosome abnormality, Gene expression, Mutation, Diagnostic markers, Prognostic markers, DNA sequencing

## Abstract

Oligodendrogliomas are typically associated with the most favorable prognosis among diffuse gliomas. However, many of the tumors progress, eventually leading to patient death. To characterize the changes associated with oligodendroglioma recurrence and progression, we analyzed two recurrent oligodendroglioma tumors upon diagnosis and after tumor relapse based on whole-genome and RNA sequencing. Relapsed tumors were diagnosed as glioblastomas with an oligodendroglioma component before the World Health Organization classification update in 2016. Both patients died within 12 months after relapse. One patient carried an inactivating *POLE* mutation leading to a clearly hypermutated progressed tumor. Strikingly, both relapsed tumors carried focal chromosomal rearrangements in *PTPRD* and *CNTNAP2* genes with associated decreased gene expression. *TP53* mutation was also detected in both patients after tumor relapse. In The Cancer Genome Atlas (TCGA) diffuse glioma cohort, PTPRD and CNTNAP2 expression decreased by tumor grade in oligodendrogliomas and PTPRD expression also in *IDH*-mutant astrocytomas. Low expression of the genes was associated with poor overall survival. Our analysis provides information about aggressive oligodendrogliomas with worse prognosis and suggests that *PTPRD* and *CNTNAP2* expression could represent an informative marker for their stratification.

## Introduction

Oligodendrogliomas are oligodendrocyte-like tumors that belong to diffuse gliomas^1, 2^ and they grow preferentially in the cortex and white matter of the cerebral hemispheres. Typical histological patterns include moderate cellularity as well as monomorphic cells with round nuclei and perinuclear halos on formalin-fixed paraffin-embedded (FFPE) sections.

The diagnostic classification of oligodendroglioma and oligoastrocytoma tumors has experienced dramatic changes in recent years. In the World Health Organization (WHO) 2007 classification, the hemizygous deletion of 1p and 19q arms (1p19q codeletion) was a hallmark alteration for oligodendrogliomas, yet it was not a prerequisite for oligodendroglioma diagnosis^2, 3^. Although oligodendrogliomas and oligoastrocytomas were graded from II to III, a grade IV glioblastoma (GBM) diagnosis with an oligodendroglioma component was possible, and this classification included oligodendroglioma-like foci and clear necrosis^2^. In 2016, the WHO updated the diffuse glioma classification by incorporating genetic alterations, especially isocitrate dehydrogenase 1 or 2 (*IDH1 or IDH2*) hotspot mutations (IDHmut) and 1p19q codeletion, as additional, or even predominant, criteria for tumor classification^1, 4^. The diagnosis of oligoastrocytomas that show histological features of both astrocytomas and oligodendrogliomas was discouraged in the WHO update, and 1p19q codeletion became a full indicator for oligodendroglioma classification. The renewed tumor grouping generated better prognostic subtypes with different genetic alteration patterns: IDH wild-type (IDHwt) astrocytomas, IDHmut astrocytomas, and oligodendrogliomas^5^. GBMs with an oligodendroglioma component were also dismissed from the classification at this point^1^, which might have been partly due to the low proportion of 1p19q codeleted cases among these GBM patients as well as the strong influence of the IDH mutation and MGMT methylation status on patient prognosis^6, 7^. In WHO classification published in 2021, oligodendroglioma became one disease entity which is further divided into two subgroups: grade 2 or 3^8^.

Among diffuse gliomas, oligodendrogliomas are typically associated with better prognosis than other tumor types^1, 2, 5^, which is partly because they respond well to treatment. They are currently graded as WHO grade 2 or 3 oligodendroglioma. Nuclear atypia and some degree of mitotic activity are compatible with grade 2 whereas significant mitotic activity, necrosis, and/or prominent microvascular proliferation indicate grade 3 status. The prognostic relevance of the grading decreases after IDH mutation and 1p19q status -based tumor classification, although grade 3 tumors are still associated with poorer overall survival than grade 2 tumors, with most patients eventually dying due to tumor recurrence^1, 2, 5, 9, 10^. Strikingly, the prognosis of IDHmut 1p19q codeleted tumors originally diagnosed as GBMs has been reported to be poor: similar to IDHwt GBMs^11^.

Capicular transcriptional repressor (*CIC*, located on 19q) and far upstream element binding protein 1 (*FUBP1*, located on 1p) gene mutations are observed in approximately 60% and 30% of oligodendrogliomas, respectively, although they are extremely rare or absent in other diffuse glioma samples^5^. These inactivating alterations together with one copy loss of 1p19q result in full inactivation of these genes. *TP53* mutations are rare and truncating *ATRX* mutations are absent among 1p19q codeleted oligodendrogliomas, whereas the majority of IDHmut astrocytomas carry these alterations^2, 5^.

Protein tyrosine phosphatase receptor type D (*PTPRD*) is a transmembrane protein phosphatase with intracytoplasmic catalytic domains (www.genecards.org)^12, 13^. It has the highest RNA expression in the brain and parathyroid gland (www.proteinatlas.org)^14^. Protein expression in the brain is detected mainly in a subpopulation of glial cells and cells in glioma tissue with a cytoplasmic and membranous staining (www.proteinatlas.org)^15^. Recurrent loss or other inactivation of *PTPRD* has been detected in different human malignancies^16–21^, and one-copy loss of *PTPRD* has also been oncogenic in p16^Ink4a^ knockout RCAS PDGFB/Nestin-tvA glioma mouse model^16^.

Contactin Associated Protein 2 (*CNTNAP2*, *CASPR2*) gene product is a transmembrane cell-adhesion protein and belongs to the neuroxin family. *CNTNAP2* is one of the largest genes in the genome and located on chromosome arm 7q, which harbors DNA copy gains in the majority of IDHwt diffuse GBMs^22, 23^. A previous publication has reported that *CNTNAP2* acts as a tumor suppressor gene in diffuse gliomas, and its alterations were detected in GBMs and an oligodendroglioma tumor^24^.

In this study, we analyzed two oligodendroglioma tumors upon diagnosis and after tumor relapse, when they were originally diagnosed as GBM with an oligodendroglioma component based on the WHO 2007 classification. Both patients survived less than twelve months after the relapse. Intragenic rearrangements were detected in both the *PTPRD* and *CNTNAP2* genes and were associated with reduced gene expression. As previous reports about these genes were before updated WHO diffuse glioma classification in 2016, we also analyzed the genetic alterations as well as DNA methylation and RNA expression levels of these genes in The Cancer Genome Atlas (TCGA) diffuse glioma cohort, which follows the current diffuse glioma classification. Our analysis revealed that *PTPRD* and *CNTNAP2* are recurrently altered in all diffuse glioma subtypes, also in oligodendroglioma, and low expression of these genes was associated with poor patient prognosis.

## Materials and methods

### Study cohort

Matched tumor samples obtained at diagnosis and after the tumor relapse were collected for this study from two patients. The study was performed in line with the principles of the Declaration of Helsinki. Approval for this study was granted by The Regional Ethics Committee of Tampere University Hospital (TAYS) (decision R07042, date 20.9.2017) and Valvira (decision V/78697/2017). The requirement for informed consent was waived by The Regional Ethics Committee of Tampere University Hospital (TAYS) because of the retrospective nature of the study. An experienced neuropathologist evaluated FFPE tumor samples and determined the histopathological type and grade according to the criteria presented by the WHO. Frozen tumor sections were stained with hematoxylin and eosin (H&E) to estimate the tumor content in the samples before DNA and RNA isolation. When frozen tumor samples were sectioned for isolation, every third section was used for RNA isolation, and the others were used for DNA isolation.

### Whole-genome sequencing

DNA was isolated from frozen samples with a QIAamp DNA Mini kit (QIAGEN) with RNase treatment. Library construction and whole-genome sequencing (WGS) with TruSeq DNA kit (Illumina) were performed at Beijing Genomics Institute (BGI), Hong Kong. Samples were sequenced with Illumina HiSeq 2000 technology using paired-end (PE90) sequencing and at least 90 Gb of clean data per sample. The pursued sequencing depth was 30 ×.

### RNA sequencing

RNA was isolated with the mirVana™ miRNA Isolation Kit (Life Technologies) so that large RNA (> 200 nucleotides) molecules were divided into a separate fraction. The library construction and sequencing of the RNA samples were performed at BGI by using Illumina HiSeq 2000 technology for sequencing. RNAs were prepared with a strand-specific 140–160 bp short insert library protocol, and at least 8 Gb of clean paired-end (PE90) data was obtained per library.

### Identification of differentially expressed genes

RNA sequencing reads were aligned with STAR version 2.7.0 against the human genome assembly GRCh38 using GENCODE version 30 annotations. Gene expression was counted using the featureCounts algorithm via the Rsubread package version 1.34.6. Read count normalization and paired differential expression between the genes before and after progression in two patients were calculated using DESeq2.

### Mutation analysis

Somatic mutations were required to have at least 3 unique supporting reads and a variant allele fraction (VAF) of 10% or higher. To filter out technical artifacts, only variants that showed a statistically significantly different allele fraction in a larger cohort of patients consisting of five similarly processed primary and relapsed astrocytomas were included in the analysis. To evaluate this, we calculated the sum of alternate and reference allele reads in each patient’s primary and secondary tumor, specified a null hypothesis that every patient has the same underlying allele fraction for the mutation, and used a chi-squared test to calculate a p-value for the probability that the observed distribution occurred by chance under the null hypothesis. Finally, any mutations that were recorded in the gnomAD v3.0 database of human germline variants were filtered out. For mutation signature analysis, the trinucleic context of the mutations was evaluated^25^, and the numbers of each substitution type were plotted.

### Copy number analysis

WGS data were aligned against the University of California, Santa Cruz (UCSC) genome browser hg38 human genome assembly^26^ using Bowtie 2 (version 2.2.9). Duplicate reads were masked using Samblaster (version 0.1.22). The genome was tiled into 1000 bp windows and the number of aligned sequencing reads was counted within each window. To exclude biases due to sequencing technology and sequence alignment, coverage log ratios were calculated relative to a whole-genome sequenced normal kidney tissue sample described in^27^. Finally, coverage log ratios were median-decimated 200-fold to suppress noise, and exported as IGV (Integrative Genomics Viewer) tracks for visual interpretation.

### Chromosomal rearrangement analysis

Chromosomal rearrangements were detected using Breakfast (commit e94e922). Rearrangements were required to be supported by at least 5 unique reads, and any rearrangements identified in over 30% of samples in a larger cohort of patients described in the Mutation analysis section were filtered out. Circos v.0.69-9 was used to visualize chromosomal rearrangements across the chromosomes. Genes that had intragenic rearrangements in both relapsed samples and altered expression between primary and relapsed samples were identified and these interesting rearrangements were more closely inspected in aligned WGS BAM files with IGV.

### DNA methylation analysis

Genome-wide DNA methylation profiling was performed using the Illumina Infinium HumanMethylation EPIC Kit. DNA methylation-based molecular classification was performed as previously described^28^. For comparison purposes, DNA-methylation profiles of the presented cases were visualized as a tSNE plot together with an in-house Heidelberg Brain Tumor Methylation Classifier cohort of 64,891 samples from different tumor entities. Heidelberg Brain Tumor Methylation Classifier is based upon 2682 central nervous system (CNS) tumors representing 82 distinct tumor methylation classes (https://www.molecularneuropathology.org)^28^. tSNE clustering was conducted on the M-values of the 10,000 most variable CpGs using the Rtsne and Rspectra packages.

### TCGA diffuse glioma data characterization

TCGA structural variants called with Delly version 0.6.3 (hg19) were downloaded from the International Cancer Genome Consortium (ICGC) Data Portal for validation analysis, and they included 41 GBM and 18 LGG cohort cases shared by TCGA and ICGC diffuse glioma cohorts. TCGA rearrangements were filtered by the fraction of rearrangement reads to the number of normal reads, which was required to be at least 0.2 either at the junction spanning reads or in minimum of 4 variant read pairs. The reported genes were inspected in the TCGA data, and intragenic rearrangements were identified.

Normalized Illumina HiSeq hg19 gene expression counts and Illumina Human Methylation 450 beta values for the TCGA-GBM and TCGA-LGG projects were acquired from the GDC legacy database through TCGAbiolinks R library v.2.18.0^29–31^. Genes that had multiple associated methylation probes were filtered based on variance higher than 0.01 between all TCGA methylation samples, including 155 GBM and 534 LGG cases. Filtered probes were clustered by the Spearman correlation distance, and the median beta value of the cluster that correlated negatively with gene expression in 503 common TCGA samples was chosen to represent the methylation of the gene. According to the WHO 2021 classification of CNS tumors, IDHwt grade 2 and 3 tumors with chromosome 7 gain and chromosome 10 loss, TERT promoter mutation or EGFR amplification were considered as IDHwt glioblastomas^8^, leaving in the cohort a total of 581 TCGA cases with expression data and 482 cases with both expression and methylation data. The significant differences in log2-transformed RNA expression between glioma subtypes or tumor grades in 581 TCGA cases (151 IDHmut oligos, 227 IDHmut astros, 203 IDHwt astros, 195 grade 2, 174 grade 3, and 212 grade 4) were calculated using a Wilcoxon rank sum test. The associations between survival and high and low log2 expression status were tested with a log-rank test and visualized with Kaplan–Meier plots using R packages survival and survminer.

Copy number alterations (CNAs) and driver mutations for the merged TCGA-LGG and GBM cohorts^32^ were downloaded from cBioPortal v.3.6.15^33, 34^. The significant differences in the number of CNAs in glioma subtypes or tumor grades were calculated using Fisher's exact test, and the differences between CNA status and log2 gene expression were calculated using the Wilcoxon rank sum test in 579 TCGA cases. CNA or driver mutation status associations with survival were estimated using the process applied for gene expression.

## Results

### Cases, clinical course, and pathological evaluation

**Figure 1:**
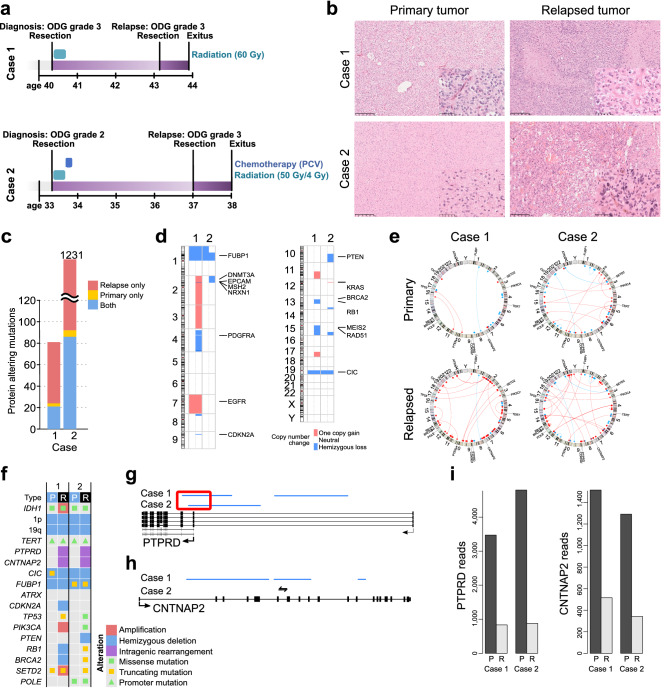
Clinical course and pathology of the cases with 1p19q codeletion. (**a**) Clinical course of the patients. ODG: oligodendroglioma, PCV: procarbazine, lomustine, and vincristine chemotherapy regimen (**b**) Representative H&E images from all the tumors. Scale bars indicate 250 µm (low magnification) and 50 µm (high magnification). (**c**) Genetic alterations detected in case 1 and hypermutator phenotype in case 2. The number of protein coding mutations detected in the primary tumor, relapsed tumor or both is represented in the figure. (**d**) New copy number alterations were detected in the relapsed tumors, more frequently in case 1. (**e**) Mainly focal intrachromosomal rearrangements accumulated in the relapsed tumors. Rearrangements are visualized with Circos plots. Rearrangements detected only in the primary or relapsed tumor are marked in red. (**f**) OncoPrint figure summarizing relevant alterations in the tumors. Both relapsed tumors harbored intragenic rearrangements in *PTPRD* and *CNTNAP2*. P: primary tumor, R: relapsed tumor. (**g**) Focal deletion at the transcription start site of *PTPRD* in both relapsed tumors. (**h**) Intragenic rearrangements detected in *CNTNAP2* after tumor relapse. Deletion was present in case 1 and inversion in case 2. (**i**) The expression of both PTPRD and CNTNAP2 was strongly decreased after relapse. Bar plot showing the number of reads in normalized samples. P: primary, R: relapse.

Case 1 was a female patient who was diagnosed with oligodendroglioma grade 3 at age 40 (Fig. 1a,b, Supplementary Table S1). Tumors were removed surgically followed by radiation treatment at 60 Gy. The tumor mass could not be fully removed based on the neurosurgeon and residual tumor was detected by postoperative MRI. The proliferation index, as measured with MIB-1 antibody staining, was 14%. Although some necrosis was detected in the primary tumor, the patient was in remission for nearly three years before relapse, when the patient was surgically operated on again. Residual tumor mass was again reported by the neurosurgeon. Tumors were diagnosed with glioblastoma at the time, although some spatial oligodendroglial features were still detected. Diagnosis was based on a strongly atypical and anaplastic cell morphology, clear GBM-like necrosis, and strong microvascular proliferation in parts of the tumor (Fig. 1b). Part of the cells corresponded to giant cell -type. Astrocytic differentiation was also detected; thus, oligoastrocytoma classification was suggested instead. The patient passed away nine months after relapse (Fig. 1a), which also suggested tumor aggressiveness.

Case 2 was a female patient who was diagnosed with oligodendroglioma grade 2 at age 33 (Fig. 1a, Supplementary Table S1). She had suffered from Hodgkin's disease four years before the glioma diagnosis and had received radiation and chemotherapy. Oligodendroglioma tumors were surgically operated on, followed by therapy with radiation (50 Gy/4 Gy) and treatment with a procarbazine, lomustine, and vincristine (PCV) regimen^35, 36^. All typical oligodendroglioma features were present in the diagnostic tissue samples with monotonic diffusively growing cells and round slightly enlarged nuclei (Fig. 1b). There were no signs of cell-rich regions, significant nuclear atypia, endovascular proliferation or necrosis. The MIB-1 proliferation index was low, with a maximum of 2%, and p53 staining was negative. Oligodendroglioma tumors relapsed at the same location 3 years and 8 months after diagnosis, and the tumor was diagnosed as GBM at the time. Oligodendroglial differentiation and associated calcification were still detected in parts of the tumor, but it also contained strongly atypical cells and geographical necrosis (Fig. 1b). Part of these cells corresponded to the giant cell type. Prominent endovascular proliferation was not detected, but the MIB-1 proliferation index was high at up to 50%. Over half of the cells were clearly positive for p53 staining. Special features mentioned in the pathological report were hyalinosis of blood vessels and cystic degeneration. Although no residual tumor was detected by the surgeon after the second surgical operation, the patient passed away 12 months after relapse.

In our WGS analysis, the primary and relapsed tumors from both cases carried the typical *IDH1* p.R132H mutation (chr2:208,248,388:G>A, VAF Case 1: 0.33 and 0.33, Case 2: 0.23 and 0.40 for primary and relapsed samples, respectively) and whole chromosome arm losses of 1p and 19q, thus confirming the oligodendroglioma diagnosis. The *TERT* promoter mutation (chr5:1,295,113:C>T, C228T), found in the majority of oligodendrogliomas, was also detected in all four tumors (VAF Case 1: 0.60 and 0.64, Case 2: 0.33 and 0.38 for primary and relapsed samples, respectively). A truncating *FUBP1* mutation (chr1:77,964,073:C>T, p.R344X) was present in both tumors of case 2 (VAF 0.25 primary, 0.90 relapsed). Furthermore, a two-nucleotide frameshift deletion (chr19:42,294,026:TC>-, p.L2288Gfs*61) was detected in *CIC* in the primary tumor of case 1 (VAF 0.50), whereas no WGS or RNAseq reads indicated this indel in the relapsed tumor sample.

Overall, oligodendroglioma hallmarks were present in primary and relapsed tumors, thus changing the diagnosis of relapsed tumors from GBM to oligodendroglioma grade 3 based on the WHO 2021 classification^8^.

### DNA methylation based classification of relapsed tumors

A DNA methylation analysis was performed as an alternative method for the classification of relapsed tumors^28^. The highest classification score for both relapsed tumors was in the DNA methylation class ‘high-grade astrocytoma’ (A IDH, HG), which includes mainly IDHmut GBMs (52.2% of cases) and anaplastic IDHmut astrocytomas (43.5% of cases)^28^. Many of the tumors in this class are secondary GBMs. The classification score for the relapsed tumor of case 1 was strong (0.999) whereas the score for the relapsed tumor for case 2 (0.893) was slightly below the threshold (0.90)^28^; thus, confident classification was not achieved. Interestingly, both tumors were located close to the oligodendroglioma sample cluster O IDH in the tSNE visualization, which was performed together with the classification (Supplementary Fig. S1). To conclude, both relapsed tumors appeared to harbor both secondary glioblastoma and oligodendroglioma features in DNA methylation analysis, which is consistent with their histopathology and genetic alterations.

### POLE mutation-induced hypermutator phenotype in case 2

When the frequencies of protein altering mutations were analyzed from the WGS data, a significantly increased mutation load was detected in case 2 (Fig. 1c). For this patient, the primary tumor already carried more mutations than case 1. After relapse, protein altering mutations increased over 12-fold (from 92 to 1145). The case exhibited a hypermutator phenotype characterized by TCT>TAT, TCG>TTG and TTT>TGT substitutions (Supplementary Fig. S2). This signature has been previously reported in DNA polymerase epsilon (*POLE*) -mutated cells in colorectal and uterine cancers^25, 37^. Correspondingly, we found a *POLE* p.A456P mutation (chr12:132,673,271:G>C, VAF 0.44 relapsed tumor) that disrupted the exonuclease domain (residues 268–471). The trinucleotide context for this mutation was GCC>GGC, suggesting that the mutation was not itself a result of the hypermutator phenotype. We also observed a p.E648K *POLE* mutation (chr12:132,668,719:G>A, VAF 0.23 relapsed tumor) in this sample, although this mutation was concordant with the TCG>TTG mutational signature and therefore likely an aftermath of the hypermutator phenotype. Interestingly, the *POLE* hypermutation signature, albeit with a lower total mutation count and only one read supporting the p.A456P mutation, was also observed in the primary tumor of this patient (VAF 0.07). Taken together, the sequencing data suggested the potential presence of a *POLE*-mutant subclone that acted as the seeding clone for progression. This hypermutator phenotype might have decreased the methylation-based tumor classification score for existing tumor classes^28^. A similar phenomenon has been previously described for DNA replication repair deficiency -related hypermutator tumors^38, 39^. In addition to *POLE*, there was a *POLE2* mutation in the relapsed sample of this patient (chr14:49,666,336:A>C, p.L190F, VAF 0.43). POLE2 is a 55-kDa isoform of the polymerase epsilon complex and has been reported to be altered in intracranial tumors^40^. The mutation was a TTA>TGA substitution and thus does not represent a typical *POLE* mutation-driven alteration. No mutations were found in the DNA repair genes *MLH1*, *MSH6*, *MSH2*, *MSH3*, *PMS1*, *PMS2* or *PCNA* in this patient.

### Copy number alterations and rearrangements increased after relapse

New chromosomal gains and losses were detected in both patients after relapse (Fig. 1d). A larger proportion of the genome involved copy number alterations in case 1 than in hypermutator case 2, consistent with previous reports^41^. Case 1 tumors harbored the gain of chromosome 7 (including *EGFR*) both before and after relapse (Fig. 1d). Similar to copy number alterations, the number of chromosomal rearrangements also increased in the relapsed tumors when compared to the matched primary tumors (Fig. 1e). A large proportion of the rearrangements were focal intrachromosomal rearrangements (Fig. 1e).

### Alterations in typical tumor suppressor genes

Although *TP53* alterations are rare among oligodendrogliomas^5^, a *TP53* mutation emerged in both cases after tumor relapse (Fig. 1f). We detected a somatic *TP53* frameshift mutation (p.V122fs, chr17:7,676,002:TG>-, VAF 0.90) with accompanying copy-neutral loss of heterozygosity in case 1 and a missense mutation (p.S127F, chr17:765,232:C>T but not the *POLE* hypermutator -type mutation, VAF 0.47) in case 2. The *TP53* p.S127F mutation has 116 entries in the COSMIC database and is classified as pathogenic with a maximum FATHMM prediction score of 1.0^42^. p53 staining was also clearly positive in the relapsed sample of case 2 (as noted above). Furthermore, other tumor suppressor genes were also altered in the relapsed tumors: case 1 harbored one copy loss of *CDKN2A*, *RB1*, and *BRCA2* genes, and case 2 harbored truncating mutations in *RB1* (chr13:48,345,108:G>T, p.E137X, VAF 0.40) and *BRCA2* (chr13:32,340,089:G>T, p.E1912X, VAF 0.50) genes as well as hemizygous deletion of *PTEN* (Fig. 1f). RB1 expression decreased 29% and CDKN2A expression increased 34% after relapse in case 1, whereas a dramatic increase in CDKN2A (11 times higher expression) was detected after relapse in case 2 (Supplementary Fig. S3, Supplementary Table S2). As CDKN2A is typically upregulated in cancers due to *RB1* inactivation^43–46^, our data suggest full inactivation of *RB1* upon relapse in case 2. No amplifications, fusions or activating mutations were detected in the growth factor receptor genes *EGFR*, *FGFR1*, *FGFR2*, *FGFR3*, *PDGFRA*, or *MET*.

### Inactivating PTPRD deletion and CNTNAP2 rearrangement in both cases after the relapse

A search for rearrangements that were present only in the relapsed and more malignant tumors detected intragenic *CNTNAP2* and *PTPRD* rearrangements in both patients. Both genes have been previously reported to act as tumor suppressor genes in diffuse glioma^16, 17, 24^. In our two patients, inactivation of *PTPRD* was caused by focal deletion of the major transcription start site after relapse (Fig. 1g). In *CNTNAP2*, intragenic deletions removing exons 2–8 and 9–12 were detected in case 1 after relapse (Fig. 1h). Both generated in-frame deletions fully or partly removing the F5/8 type C, laminin G-like 1–2, EGF-like 1, and fibrinogen C-terminal domains. These domains are extracellular, and their loss likely alters ligand interactions in the extracellular space. Due to protein dimerization, intragenic rearrangement in one allele is likely to alter the function of wild-type CNTNAP2^47^. Several observed deletions might be related to the gain of this gene in case 1 (Fig. 1d). In case 2, intragenic inversion was detected in *CNTNAP2* after relapse in the intronic region (chr7:147,218,816–147,293,814) (Fig. 1h), which includes several conserved predicted enhancers^26, 48^. The RNA expression levels of both PTPRD and CNTNAP2 were strongly reduced upon relapse (Fig. 1i, Supplementary Table S2).

### Low PTPRD and CNTNAP2 expression is associated with higher tumor grade and poor patient survival

To further study the clinical significance of PTPRD and CNTNAP2 in diffuse gliomas with the current WHO tumor classification, we analyzed TCGA diffuse glioma data for chromosomal rearrangements, CNAs, DNA methylation, and RNA expression levels of these genes.

**Figure 2:**
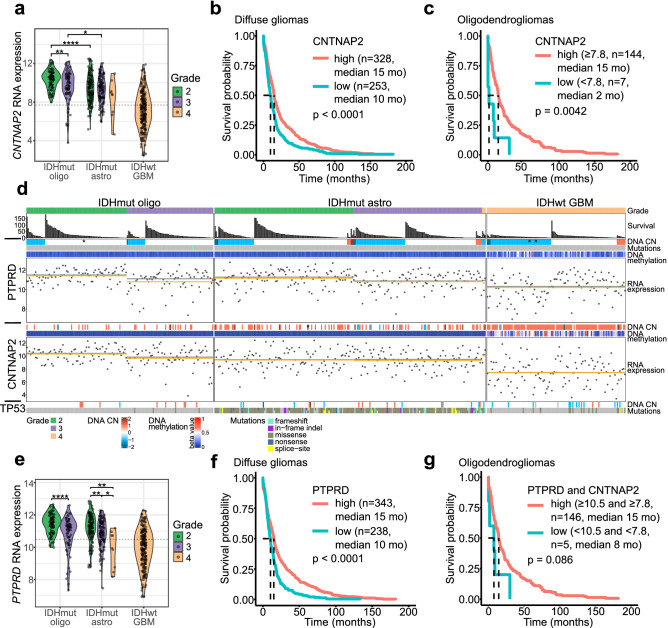
CNTNAP2 and PTPRD expression is associated with tumor aggressiveness and poorer patient survival. (**a**) CNTNAP2 expression in 581 diffuse glioma cases including 151 IDHmut oligodendrogliomas (oligo), 227 IDHmut astrocytomas (astro) and 203 IDHwt glioblastomas. Statistical significance was calculated between tumor grades in each subtype and between different tumor types with the same grade. *p < 0.05, **p < 0.01, and ****p < 0.0001 based on the Wilcoxon rank-sum test. Selected cutoff 7.8 for high and low expression of CNTNAP2 is marked with a dashed line. (**b**, **c**) Low CNTNAP2 expression was associated with poor overall survival in both b) the whole diffuse glioma cohort (581 cases, log-rank test) and c) within oligodendrogliomas (151 cases, log-rank test). (**d**) Summary of *PTPRD* and *CNTNAP2* alterations and expression levels in diffuse glioma (482 cases with DNA methylation, DNA copy number and RNA expression data). Both DNA methylation and decreased copy number (CN) are associated with lower PTPRD expression, whereas CNTNAP2 expression was only linked to DNA methylation. Tumors harboring intragenic rearrangements of *PTPRD* or *CNTNAP2* are indicated with an asterisk. The mean and median RNA expression are presented in yellow and gray, respectively. (**e**) PTPRD expression decreases by tumor grade in oligodendrogliomas and IDHmut astrocytomas but does not differ between tumor subtypes (581 cases, Wilcoxon rank sum test). Dashed line indicates the selected cutoff 10.5 for high and low expression of PTPRD. (**f**) Low PTPRD expression is associated with poor overall survival in diffuse gliomas (581 cases, log-rank test). (**g**) Low survival rates detected in oligodendroglioma patients with low expression of both PTPRD and CNTNAP2 (151 cases) using cutoffs of 7.8 for CNTNAP2 and 10.5 for PTPRD.

In the TCGA cohort of 581 diffuse gliomas (Supplementary Table S3), the lowest CNTNAP2 expression was observed in IDHwt glioblastoma tumors (Fig. 2a). An association with tumor grade was detected within oligodendrogliomas but not among IDHmut astrocytomas (Fig. 2a). Low CNTNAP2 expression (below 8.75, mean expression in the whole cohort) was also significantly associated with poorer prognosis in diffuse gliomas (p < 0.0001, log-rank test) (Fig. 2b). Strikingly, low CNTNAP2 expression (below 7.8) was detected in a subpopulation of oligodendrogliomas (Fig. 2a) and associated with poor patient survival (p = 0.0042, log-rank test) (Fig. 2c). A similar association with survival was not observed within IDHwt glioblastomas or IDHmut astrocytomas (Supplementary Fig. S4).

In the TCGA rearrangement cohort of 59 diffuse gliomas, intragenic *CNTNAP2* rearrangements were detected in four tumors, including three IDHwt glioblastomas and one IDHmut astrocytoma (Supplementary Table S4, Supplementary Fig. S5). Although low CNTNAP2 expression was not generally associated with intragenic rearrangements of the gene, low gene expression was detected in one case and the patients carrying these tumors showed poor survival rates. Furthermore, DNA copy number losses were rare in *CNTNAP2* while gains were recurrently detected in the TCGA cohort of 482 diffuse gliomas, especially in IDHwt glioblastomas (Fig. 2d), which is understandable because the gain of chromosome 7 (or part of it) is common in these tumors^23^. *CNTNAP2* is located on chr7q35. Higher CNTNAP2 expression was not associated with increased DNA copy number (Supplementary Fig. S5). However, the *CNTNAP2* gene was methylated especially in IDHwt samples with *CNTNAP2* gain (p < 2.2 × 10^–16^, 32% and 0.5% samples methylated within IDHwt samples with *CNTNAP2* gain and other samples, respectively, n = 482) (Fig. 2d), and DNA methylation was linked to lower CNTNAP2 expression (Supplementary Fig. S6).

Lower PTPRD expression was significantly associated with higher tumor grade in oligodendrogliomas and IDHmut astrocytomas (p < 0.05 between all grades, Wilcoxon rank sum test, 581 diffuse glioma cases) (Fig. 2e, Supplementary Table S3). No significant differences were detected between the tumor subtypes within the same grade (Fig. 2e). Furthermore, low PTPRD expression (below 10.67, mean expression in the whole cohort) was significantly associated with poorer overall survival within diffuse gliomas (p < 0.0001, log-rank test) (Fig. 2f). A subpopulation of oligodendrogliomas showed low expression of PTPRD (below 10.5) (Fig. 2e). Low PTPRD expression was associated with poorer survival in diffuse gliomas also using the cutoff 10.5 (Supplementary Fig. S7). A similar trend was detected when the analysis was performed for oligodendrogliomas and IDHmut astrocytomas, although the results were not significant (Supplementary Fig. S7).

In the TCGA rearrangement cohort of 59 diffuse gliomas, there were four samples harboring intragenic *PTPRD* rearrangements (Supplementary Table S4): one grade 2 oligodendroglioma and three grade 4 IDHwt glioblastomas. Two out of the four samples with expression data, including the oligodendroglioma tumor, had low expression when compared to the samples with the same tumor type and grade (Supplementary Fig. S8). Survival rates were poor in all the patients carrying these rearrangements (Fig. 2d, Supplementary Table S4).

*PTPRD* DNA copy number losses, which were mainly hemizygous losses, were recurrently detected in all the tumor types (Fig. 2d) within 581 diffuse glioma cases and associated with reduced PTPRD expression (Supplementary Fig. S8). They were also linked to reduced overall survival (Supplementary Fig. S8). *PTPRD* DNA methylation was especially detected in IDHwt glioblastomas irrespective of the copy number status (Fig. 2d), and it was associated with reduced PTPRD expression (Supplementary Fig. S9).

Because our patients carried *TP53* mutations after relapse, we also analyzed the association between *TP53* driver mutation status and low expression of CNTNAP2 or PTPRD in the TCGA cohort. TP53 driver mutations were detected in 89% of the IDHmut astrocytomas, which is consistent with previous reports^5^, and they were also present in a subpopulation of oligodendrogliomas and IDHwt glioblastomas (Fig. 2d). *TP53* driver mutations were not associated with low PTPRD or CNTNAP2 expression (Fig. 2d) or overall survival (Supplementary Fig. S10) in any of the diffuse glioma subtypes.

Taken together, the data support previous reports about the tumor suppressor role of PTPRD and CNTNAP2 in diffuse gliomas and show that low CNTNAP2 expression is associated with poor overall survival especially in oligodendrogliomas. Although statistical significance was not reached for PTPRD in oligodendrogliomas, poor survival rates were detected in cases with low PTPRD expression. The expression of CNTNAP2 and PTPRD showed a moderate positive correlation in the TCGA diffuse glioma cohort, and there were cases that had low expression of both genes (Supplementary Fig. S11). We thus analyzed them together. The survival rates were poor for oligodendroglioma cases with low expression of both PTPRD and CNTNAP2 (median 8 months, maximum 30 months) (Fig. 2g). Significantly poorer survival was observed for these cases in the whole diffuse glioma cohort (p < 0.0001, log-rank test) (Supplementary Fig. S12), but a similar trend was not detected in the IDHmut astrocytomas or IDHwt glioblastomas (Supplementary Fig. S12).

## Discussion

Our analysis of primary and recurrent oligodendrogliomas revealed rearrangements in *CNTNAP2* and *PTPRD* genes in the recurrencies, which were originally diagnosed as secondary GBMs because they showed histological signs of tumor progression. Both *CNTNAP2* and *PTPRD* have been previously reported to act as tumor suppressor genes in diffuse glioma: One copy loss of *PTPRD* has been oncogenic in the p16^Ink4a^ knockout RCAS PDGFB/Nestin-tvA glioma mouse model^16^, which is prone to generating oligodendroglioma tumors^49^. The oncogenicity was not observed when both copies of the gene were deleted^16^, which is consistent with the typical loss or inactivation of only one copy of *PTPRD* in human malignancies^16–21^. Similarly, recurrent *CNTNAP2* alterations were reported in GBMs and an oligodendroglioma, often leading to reduced protein expression, CNTNAP2 expression was associated with poor overall survival both in univariate and multivariate analyses, and its increased expression led to decreased proliferation and increased apoptosis in GBM cells^24^. The association with patient survival was not linked to lower tumor purity in samples with higher CNTNAP2 expression^24^, which also suggests tumor-specific effects of the protein.

As both *CNTNAP2* and *PTPRD* analyses predated the updated WHO diffuse glioma classification, we analyzed these further in the TCGA cohort with updated tumor classification based on IDH mutation and 1p19q co-deletion. The results revealed that lower expression of the genes was associated with higher tumor grade in oligodendrogliomas and poorer patient prognosis. Both genes were also recurrently targeted by genetic alterations and/or DNA methylation in all diffuse glioma tumor types. Importantly, significant or close to significant survival associations were also detected in the oligodendrogliomas, revealing a patient group with poorer prognosis. Maximum time-lapse from diagnosis to death was 30 months for oligodendroglioma cases with low expression of CNTNAP2 or both CNTNAP2 and PTPRD. Additionally, in our cases, *TP53* mutations were detected in both patients after relapse and, based on the inactivating *RB1* mutation and increased CDKN2A expression, the RB1 pathway appeared to be inactivated in the relapsed tumor of case 2, potentially affecting proliferation. In the TCGA analysis, *TP53* driver mutations were not detected along with low CNTNAP2 or PTPRD expression in oligodendrogliomas.

Our results suggest that the *POLE*-mutant subclone is the seeding clone for tumor progression in case 2. It is likely that *POLE* mutation has an influence on tumor characteristics. Giant multinucleated cells, abundant immune infiltration, and atypical mitotic figures were present in the relapsed tumor of case 2, which is similar to previous reports^41^. However, inactivating *POLE* mutations and a hypermutator phenotype have been linked to increased immune response and better prognosis in GBM, anaplastic astrocytoma, pediatric brain tumors, and other malignancies^41, 50, 51^, including a case with an exceptionally good prognosis^50^. Better prognosis of these patients was not caused by checkpoint inhibition as these patients did not receive it. *POLE* mutations and the hypermutator phenotype are thus less likely to be the main drivers of increased malignancy and an associated poor prognosis in the relapsed tumors.

The general number of DNA copy number alterations and rearrangements increased in the relapsed tumors. Both patients received radiation after the primary brain tumor diagnosis, which might have had an effect. However, these patients were not treated with temozolomide, so it is not influencing the observed relapse-related changes in genetic alterations or gene expression patterns.

Both PTPRD and CNTNAP2 are transmembrane proteins that have been linked to myelination and oligodendrocyte development. CNTNAP2 protein is needed for proper potassium channel clustering, axonal growth, and neuronal myelination during development, and several *CNTNAP2* variants have been associated with different neurodevelopmental disorders^22, 47, 52–54^. Loss of one functional allele is sufficient to disrupt axonal growth^55^. Although functional defects of CNTNAP2 are typically linked to neurons, the gene is expressed in oligodendrocyte precursor cells, which also drive the myelination process after their differentiation^56, 57^, and oligodendrocyte counts are decreased in *CNTNAP2* mutant juvenile mice^53^. Similarly, PTPRD is expressed in postnatal oligodendrocytes and is involved in the myelination process of these cells^58^, and glial cell differentiation appears to be shifted from oligodendroglial differentiation to increased astroglial differentiation after hemizygous loss of *PTPRD*^16^. *PTPRD* is on the same chromosome arm as *CDKN2A*, which is recurrently homozygously deleted in high-grade diffuse gliomas. However, the hemizygous loss of *PTPRD* was not associated with *CDKN2A* inactivation in our cases, thus decreasing the possibility of a bystander hit. Instead, *PTPRD* was specifically inactivated with focal intragenic deletion, which led to clear downregulation of gene expression. As the hemizygous loss or inactivation of *PTPRD* leads to increased STAT3 phosphorylation^16, 59, 60^, tumor suppressor role of PTPRD is mediated at least partly by the dephosphorylation of this relevant oncoprotein. PTPRD has also other relevant targets, such as aurora kinase A (AURKA), which may also mediate its tumor suppressive functions^61^. Moreover, CNTNAP2 protein is able to interact with CNTN2^62^, which has been linked to glioma stem cell proliferation and shows higher expression levels in oligodendrogliomas^63–65^, and EPB41L3 (alias DAL1, 4.1B)^66^, which is reported to act as a tumor suppressor gene in glioma^67–69^.

Overall, our results reveal that although oligodendrogliomas are generally associated with better survival than other subtypes of diffuse glioma, some oligodendrogliomas are aggressive and show poor survival rates. PTPRD and CNTNAP2, which are recurrently altered in diffuse gliomas and show decreased expression in a subpopulation of patients, could be informative for the stratification of these aggressive oligodendrogliomas by analyzing their inactivating alterations and gene expression levels. Our results can also improve the understanding of the tumor evolution especially when patients have been treated with radiation.

## Supplementary Information

Supplementary Figures.

Supplementary Tables.

## Data Availability

Normalized gene expression counts are available in GEO with an accession number GSE196707. The raw data from datasets generated during the current study are not publicly available due lack of patient consent that separately gives permission to this, but the corresponding author can be contacted upon reasonable request. Normalized Illumina HiSeq gene expression counts and Illumina Human Methylation 450 beta values for the TCGA-GBM and TCGA-LGG projects used in this study can be retrieved directly from the public GDC legacy data portal (https://portal.gdc.cancer.gov/legacy-archive/search/f) or using the R library (https://bioconductor.org/packages/release/bioc/html/TCGAbiolinks.html). Copy number alterations and driver mutations in TCGA-GBM and TCGA-LGG cohorts can be accessed through cBioPortal (https://www.cbioportal.org/study/summary?id=lgggbm_tcga_pub). TCGA-GBM and TCGA-LGG structural variants from the DKFZ/EMBL variant calling pipeline can be retrieved from the ICGC data portal (https://dcc.icgc.org/repositories).
