# Supplementary material for: PTPRD and CNTNAP2 as markers of tumor aggressiveness in oligodendrogliomas
Source: Sci Rep. 2022 Aug 18;12:14083. doi: 10.1038/s41598-022-14977-2 (PMC9388569; doi:10.1038/s41598-022-14977-2)
Supplement: Supplementary file 1 — Supplementary Figures. [file 41598_2022_14977_MOESM1_ESM.pdf]

# PTPRD and CNTNAP2 as markers of tumor aggressiveness in oligodendrogliomas

Kirsi J. Rautajoki, Serafiina Jaatinen, Aliisa M. Tiihonen, Matti Annala, Elisa M. Vuorinen, Anni Kivinen, Minna J.Rauhala, Kendra K. Maass, Kristian W. Pajtler, Olli Yli-Harja, Pauli Helén, Joonas Haapasalo, Hannu Haapasalo, Wei Zhang, Matti Nykter

## Figures S1-S12

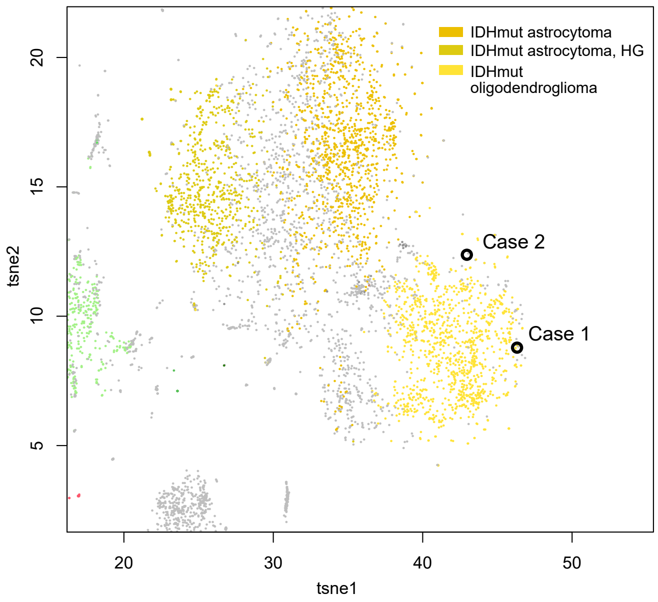


Figure S1. DNA methylation based clustering of relapsed tumors in tSNE visualization. Zoom in highlights IDH mutant astrocytoma, IDH mutant astrocytoma and IDH oligodendroglioma clusters. HG: high-grade.

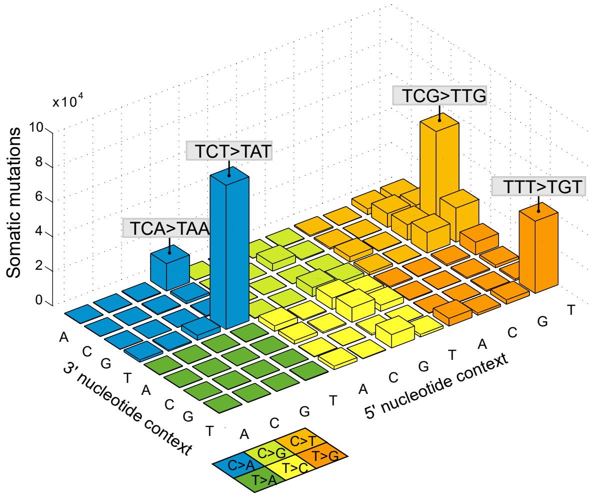


Figure S2. Mutations represented mainly TCT>TAT, TCG>TTG and TTT>TGT substitutions, which are typical for tumors with POLE deficiency.

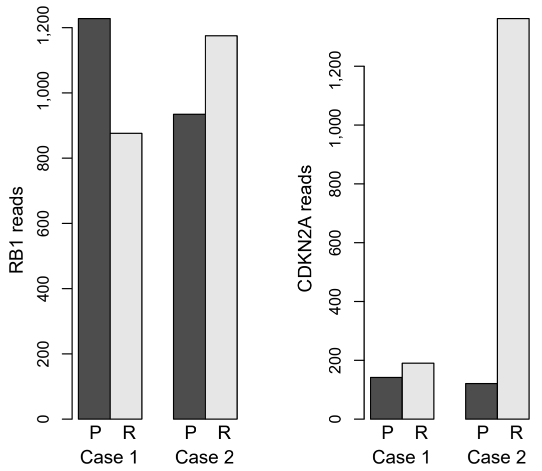


Figure S3. CDKN2A and RB1 expression in the cases. Bar plot showing the number of reads in normalized samples. P: primary, R:relapse.

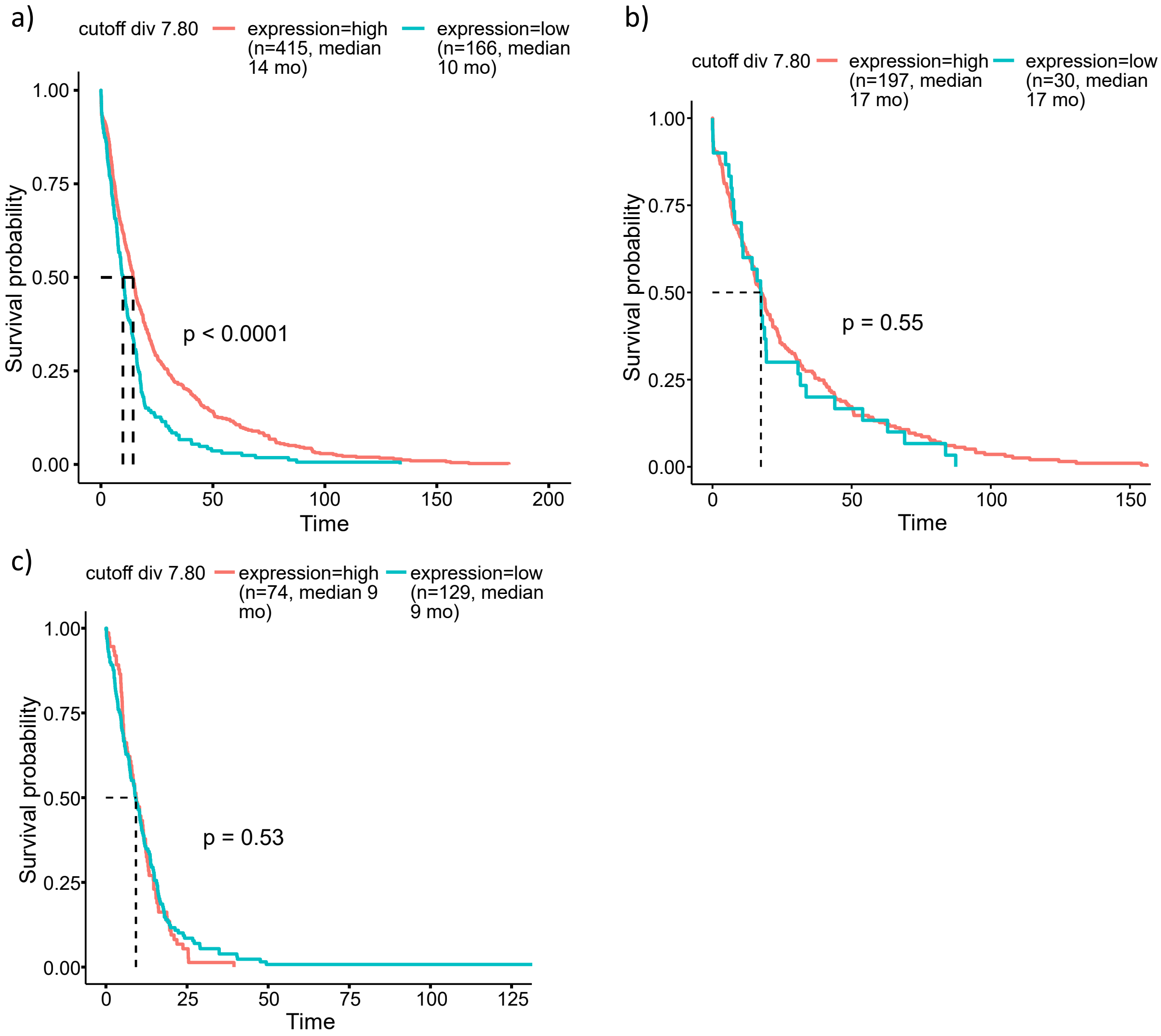


Figure S4. The association of CNTNAP2 expression with patient survival in a) the whole diffuse glioma cohort, b) IDHmut astrocytomas, and c) IDHwt glioblastomas. Time is in months.

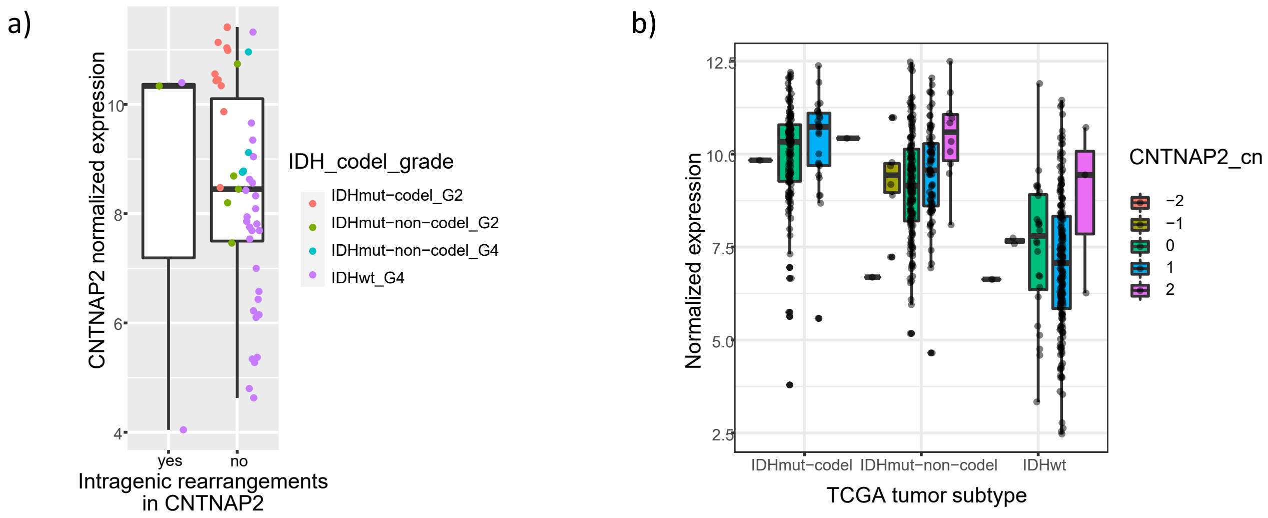


Figure S5. Relation of alterations to expression in CNTNAP2. a) Rearrangements were not generally associated with CNTNAP2 expression, but one case with an intragenic *CNTNAP2* rearrangement shows also very low CNTNAP2 expression. Expression data was not available from one case who harboured CNTNAP2 rearrangement. b) Loss of CNTNAP2 was not associated with decreased CNTNAP2 expression.

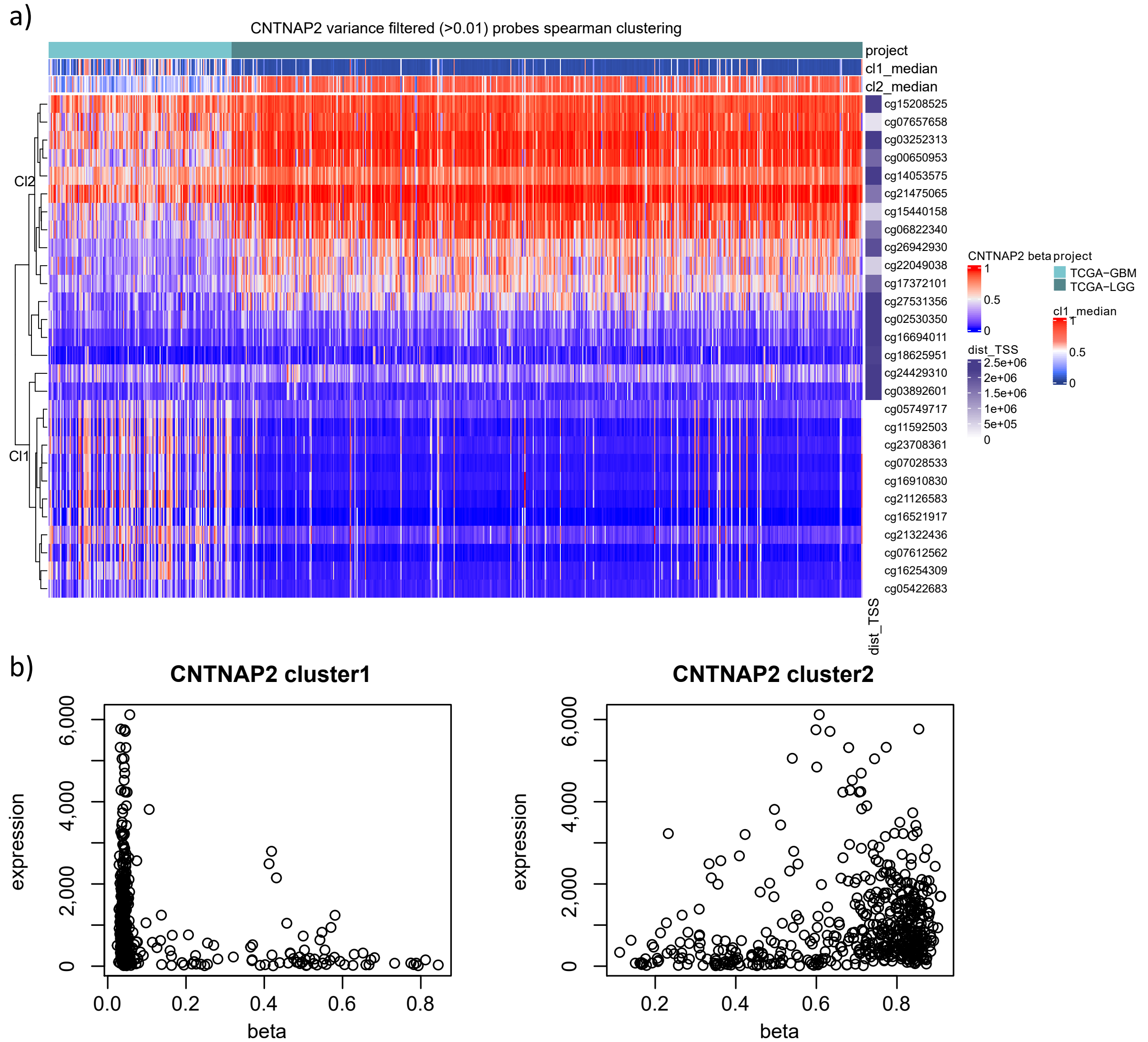


Figure S6. DNA methylation is linked to CNTNAP2 expression. a) Heatmap showing the beta values for variable DNA methylation microarray probes linked to *CNTNAP2* gene. Probes in the upper part of the figure are included in cluster2 (Cl2) and those at the lower part are included in cluster1 (Cl1). The heatmap is created with a R package ComplexHeatmap v. 2.6.2. (https://github.com/jokergoo/ComplexHeatmap). b) Scatter plot showing the correlation between expression and DNA methylation (correlation coefficients: cluster1 Pearson -0.27, Spearman -0.35; cluster2 Pearson 0.20, Spearman 0.30). Probes in cluster1 were selected for further analysis.

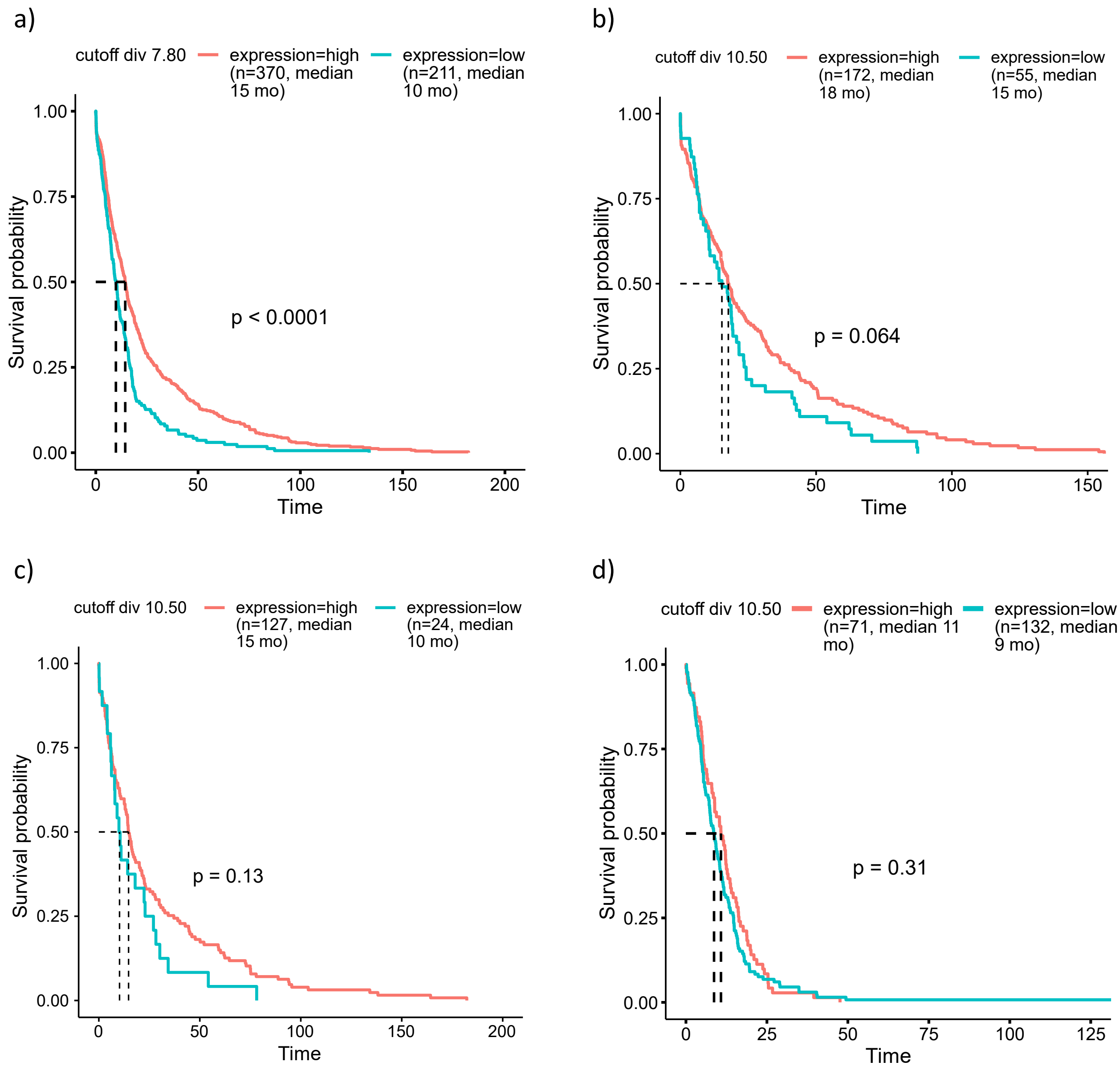


Figure S7. The association of PTPRD expression with survival in a) the whole diffuse glioma cohort, b) IDHmut astrocytomas, c) IDHmut oligodendrogliomas, and d) IDHwt glioblastomas. Time is in months.

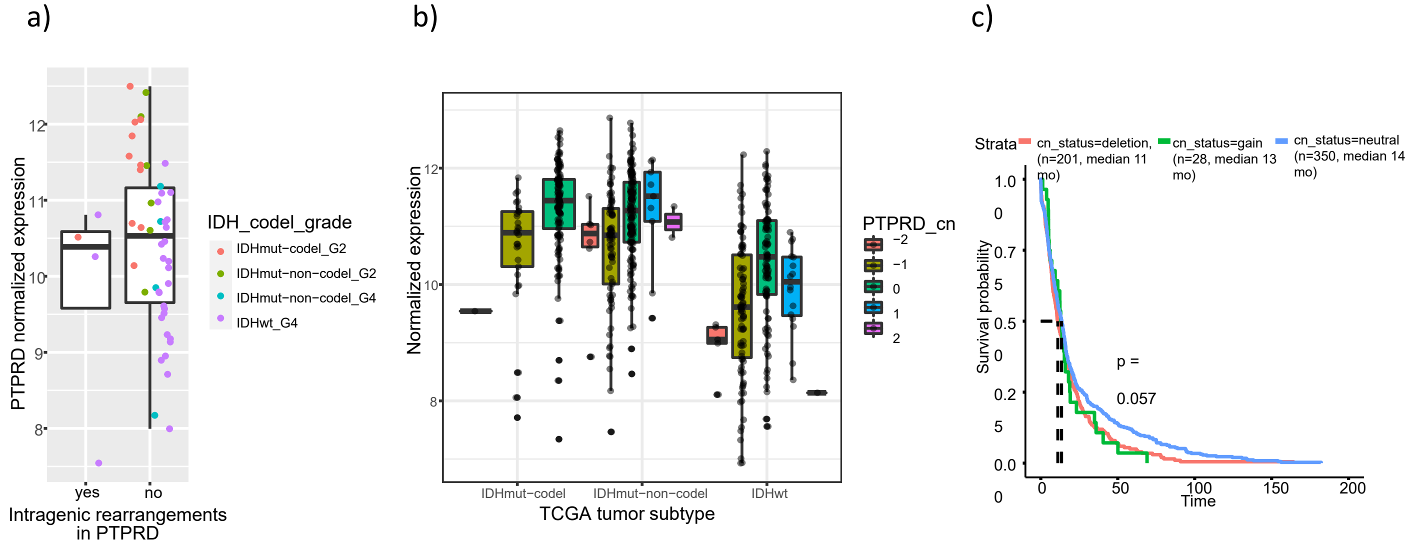


Figure S8. Alterations in PTPRD associated with survival. a) Rearrangements were not generally associated with PTPRD expression, but two cases with an intragenic *PTPRD* rearrangement have low PTPRD expression in comparison to the other cases with the same tumor type (oligodendroglioma or IDHwt glioblastoma). Loss of *PTPRD* was associated with b) decreased gene expression and c) decreased survival. Time is in months.

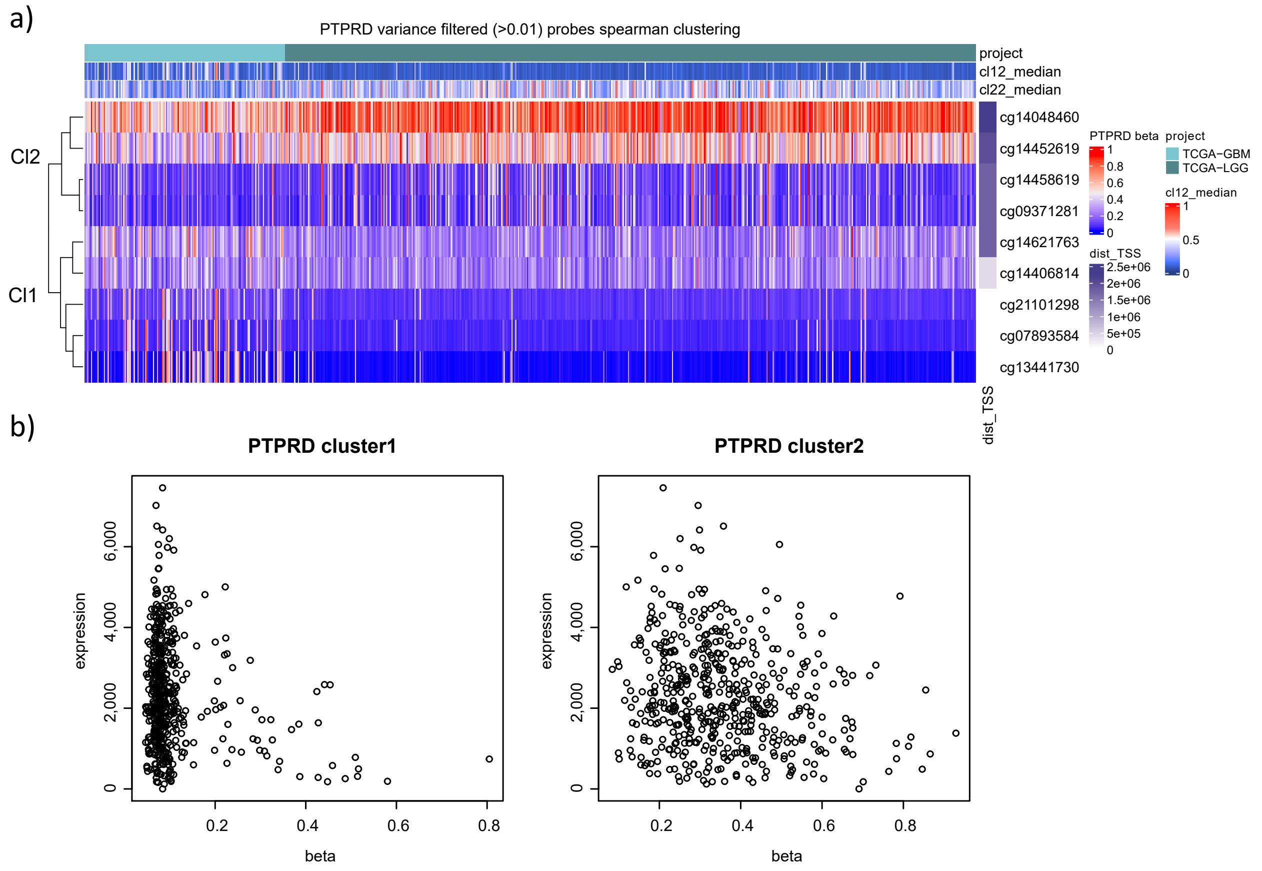


Figure S9. DNA methylation is linked to PTPRD expression. a) Heatmap showing the beta values for DNA methylation microarray probes linked to *PTPRD* gene. Probes in the upper part of the figure are included in cluster2 (Cl2) and those at the lower part are included in cluster1 (Cl1). The heatmap is created with a R package ComplexHeatmap v. 2.6.2. (https://github.com/jokergoo/ComplexHeatmap). b) Scatter plot showing the correlation between expression and DNA methylation (correlation coefficients: cluster1 Pearson -0.21, Spearman -0.11; cluster2 Pearson -0.18, Spearman -0.17). Probes in cluster1 were selected for further analysis.

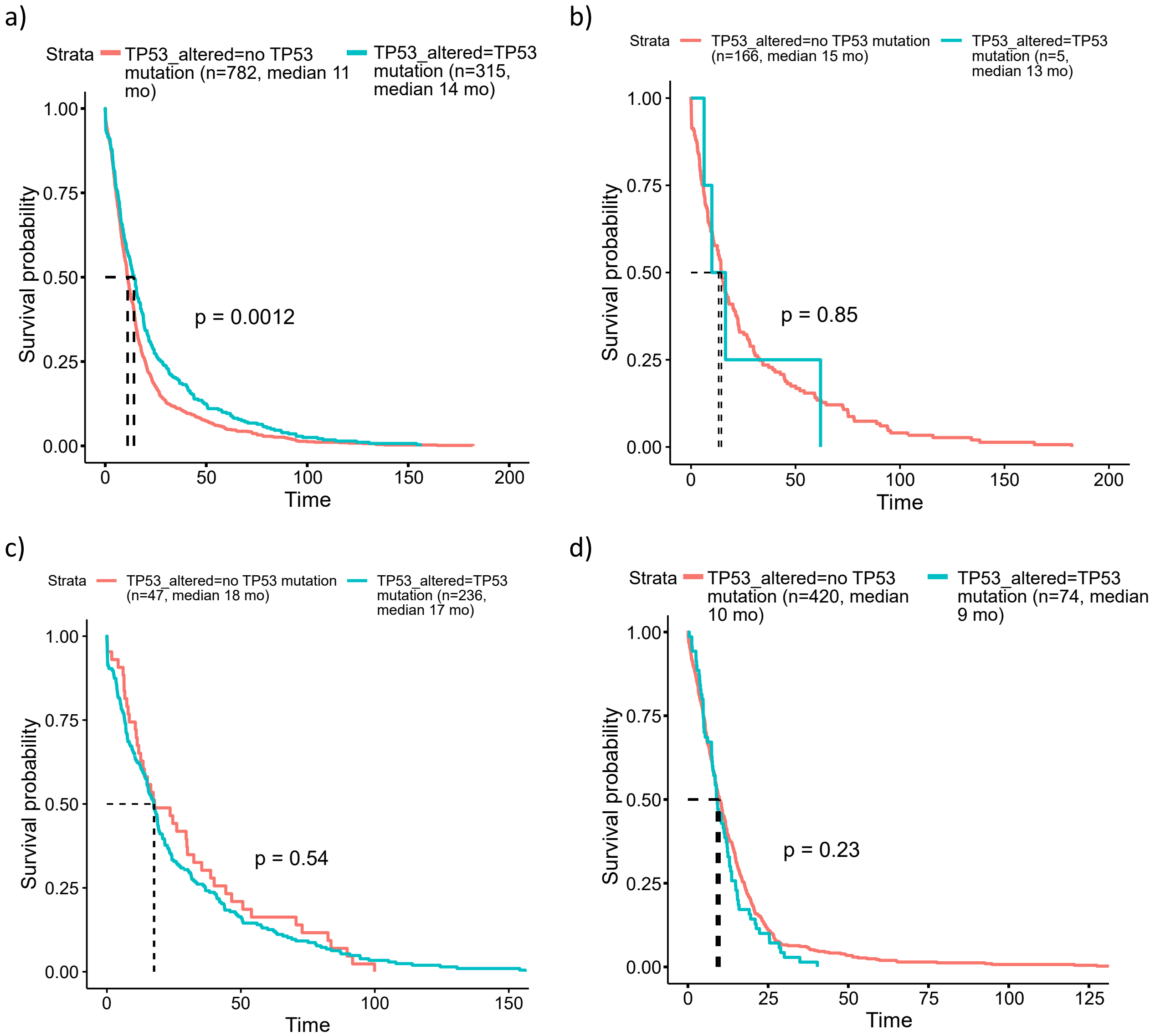


Figure S10. TP53 driver mutations were not associated with survival in any of the diffuse glioma subtypes. Survival rates in a) the whole diffuse glioma cohort, b) oligodendrogliomas, c) IDHmut astrocytomas, and d) IDHwt glioblastomas. Time is in months.

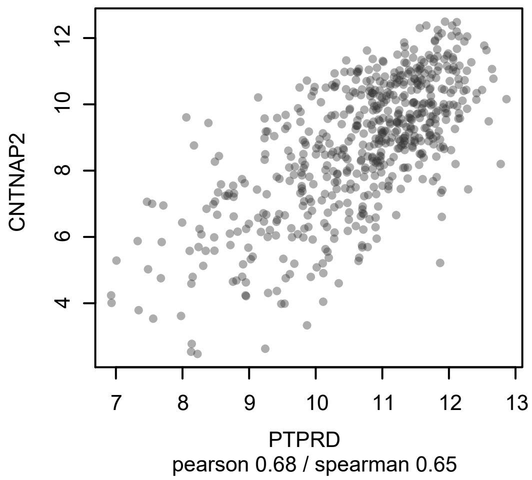


Figure S11. Correlation between PTPRD and CNTNAP2 in the TCGA diffuse glioma cohort.

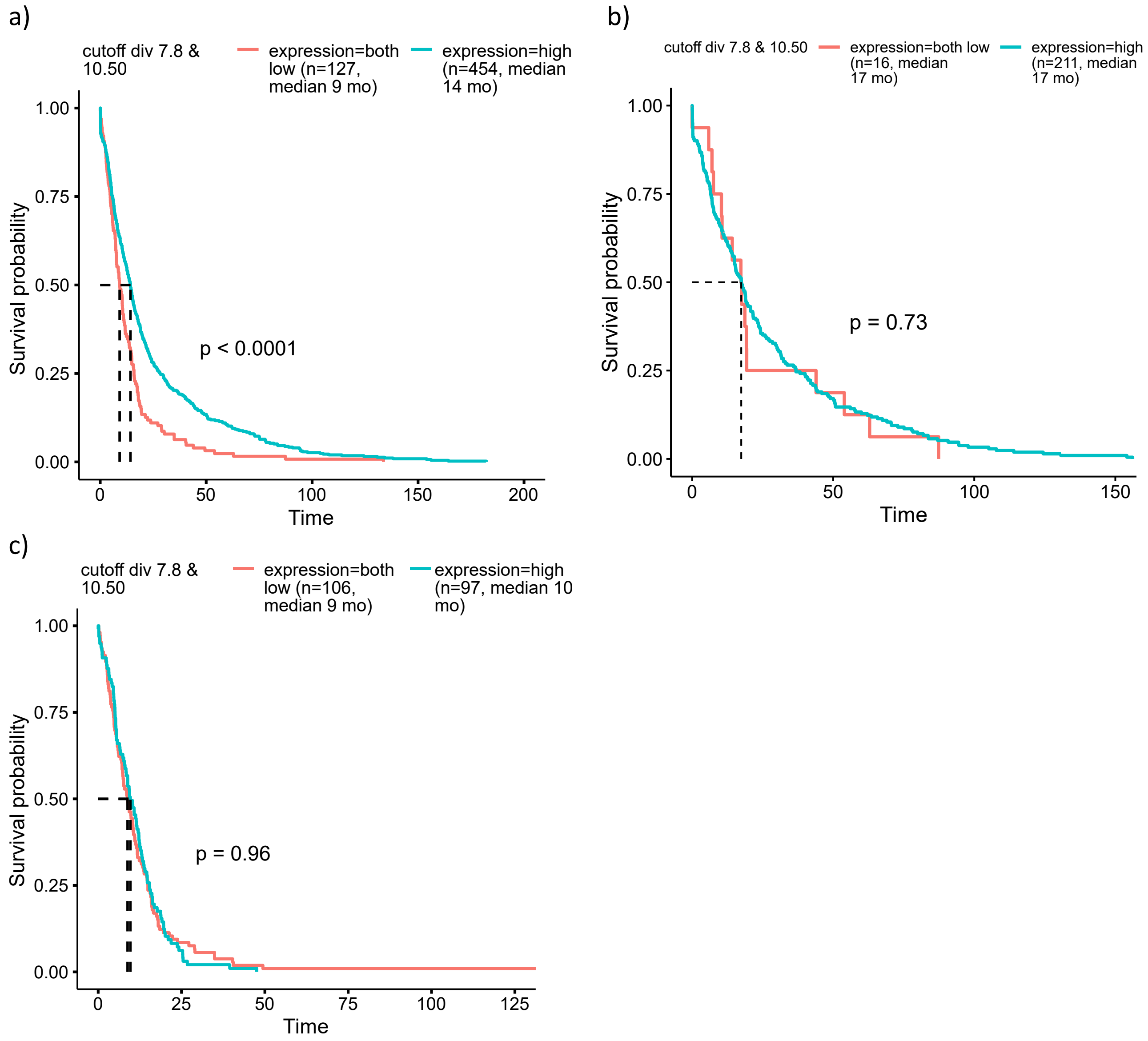


Figure S12. The association of the low expression of both CNTNAP2 and PTPRD with survival in a) the whole diffuse glioma cohort, b) IDHmut astrocytomas, and c) IDHwt glioblastomas. Time is in months.
